# Association between Serum Free Fatty Acids and Clinical and Laboratory Parameters in Acute Heart Failure Patients

**DOI:** 10.3390/biomedicines11123197

**Published:** 2023-12-01

**Authors:** Iva Klobučar, Helga Hinteregger, Margarete Lechleitner, Matias Trbušić, Gudrun Pregartner, Andrea Berghold, Wolfgang Sattler, Saša Frank, Vesna Degoricija

**Affiliations:** 1 Department of Cardiology, Sisters of Charity University Hospital Centre, 10000 Zagreb, Croatia; iva.klobucar@gmail.com (I.K.); matias.trbusic@gmail.com (M.T.); 2 Gottfried Schatz Research Center, Molecular Biology and Biochemistry, Medical University of Graz, 8010 Graz, Austria; helga.reicher@medunigraz.at (H.H.); margarete.lechleitner@medunigraz.at (M.L.); wolfgang.sattler@medunigraz.at (W.S.); 3 School of Medicine, University of Zagreb, 10000 Zagreb, Croatia; vesna.degoricija@mef.hr; 4 Institute for Medical Informatics, Statistics and Documentation, Medical University of Graz, 8036 Graz, Austria; gudrun.pregartner@medunigraz.at (G.P.); andrea.berghold@medunigraz.at (A.B.); 5 BioTechMed-Graz, 8010 Graz, Austria; 6 Department of Medicine, Sisters of Charity University Hospital Centre, 10000 Zagreb, Croatia

**Keywords:** acute heart failure, free fatty acids, gas chromatography

## Abstract

Very little is known about the association between individual serum free fatty acids (FFAs) and clinical and laboratory parameters (indicators of heart failure severity) in acute heart failure (AHF) patients. Here, the baseline serum levels of FFAs, 16:0 (palmitic acid), 16:1 (palmitoleic acid), 18:0 (stearic acid), 18:1 (oleic acid), 18:2 (linoleic acid), 18:3 (alpha-linolenic acid or gamma-linolenic acid), 20:4 (arachidonic acid), 20:5 (eicosapentaenoic acid), and 22:6 (docosahexaenoic acid), were determined in 304 AHF patients (94.7% belonged to New York Heart Association functional class IV) using gas chromatography. Spearman correlation coefficients were used to examine the associations between the individual and total (the sum of all FFAs) FFAs and clinical and laboratory parameters. After applying a Bonferroni correction to correct for multiple testing, the total FFAs, as well as the individual FFAs (except FFAs 18:0, 20:5, and 22:6), were found to be significantly positively correlated with serum albumin. Only a few additional associations were found: FFA 16:0 was significantly negatively correlated with systolic pulmonary artery pressure, FFA 18:3 was significantly negatively correlated with C-reactive protein and body mass index, and FFA 20:4 was significantly negatively correlated with blood urea nitrogen. Based on our results, we conclude that in patients with severe AHF, individual and total serum FFAs are slightly associated with established laboratory and clinical parameters, which are indicators of heart failure severity.

## 1. Introduction

Heart failure (HF) is the final stage of various cardiovascular diseases and therefore a frequent cause of disability and death worldwide [1]. The failing heart exhibits an altered structure and consequently an impaired function, resulting in the diminished perfusion of metabolizing tissues [2,3,4]. Acute heart failure (AHF) is characterized by either the rapid onset or worsening of the signs and symptoms of HF [3]. 

Decreased peripheral perfusion in HF triggers compensatory mechanisms, which then further deteriorate the underlying pathophysiology, leading to disease progression and increased severity [5]. More specifically, the under-perfusion of kidneys stimulates the renin–angiotensin system (RAS) and, in turn, aldosterone and norepinephrine secretion. This leads to increased renal sodium and water retention, as well as increased systemic blood pressure, which further weakens the function of the failing heart [6]. Left ventricular dysfunction and heart wall distension trigger an elevation in natriuretic peptides, which only partially counteract the effects of activated RAS, aldosterone, and increased sympathetic tone. Peripheral venous congestion (a consequence of right-sided HF) and hypoperfusion worsen renal and liver function and also diminish the motility of the gut, resulting in an altered composition of the gut microbiota and leading to gut inflammation and diminished nutrient absorption, as well as a systemic inflammatory response [7,8,9,10]. The latter is primarily caused by the compromised barrier function of the edematous and inflamed intestine and consequently the increased translocation of bacterial toxins into the circulation, as well as the congestion-induced activation of venous endothelial cells [11,12].

Increased serum levels of catecholamines, natriuretic peptides, and inflammatory cytokines are strong inducers of adipose tissue lipolysis, the hallmark of catabolic dominance and a major source of serum free fatty acids (FFAs) [13,14]. Serum FFAs are also generated by the serum-lipase-mediated hydrolysis of lipids in circulating lipoproteins, a process regulated by nutritional state, hormones, and inflammatory cytokines [15]. High serum levels of FFAs are potent inducers of insulin resistance, a frequent pathophysiological component of HF, as well as oxidative stress, inflammatory response, and endothelial dysfunction, with the latter being an inherent feature of vascular pathophysiology in HF [16,17,18]. In HF, an increased uptake of FFAs by cardiomyocytes, secondary to elevated FFA serum levels, leads to the accumulation of FFA-derived cardiotoxic intermediates that, together with diminished glucose and increased FFA utilization for cardiac ATP production, deteriorate the function of the failing heart [19,20,21]. Pharmacological interventions capable of reducing FFAs and increasing glucose utilization for energy production improve cardiac function and prognosis in patients with chronic HF (CHF) [18,22,23,24].

Total serum FFA levels are higher in patients with CHF compared with healthy controls, and high levels of total FFAs have been found to be associated with increased mortality in AHF patients [25,26,27]. Several studies have examined the association between various individual serum FFAs and mortality in AHF patients [28,29,30,31], but none of them have reported on the association of studied FFAs with patients’ clinical and laboratory characteristics. Therefore, in the present study, we determined the serum levels of FFAs 16:0 (palmitic acid), 16:1 (palmitoleic acid), 18:0 (stearic acid), 18:1 (oleic acid), 18:2 (linoleic acid), 18:3 (alpha-linolenic acid or gamma-linolenic acid), 20:4 (arachidonic acid), 20:5 (eicosapentaenoic acid), and 22:6 (docosahexaenoic acid) and examined their association with clinical and laboratory parameters, established indicators of HF severity, in AHF patients.

## 2. Materials and Methods

### 2.1. Study Design and Patients

The AHF study was a prospective observational study conducted at the Sisters of Charity University Hospital Centre, Zagreb, Croatia, from March 2018 to January 2021, enrolling consecutive adult Caucasian patients who presented to the Emergency Department with signs and symptoms of AHF consequently requiring hospitalization. The coexistence of any case of acute infection, chronic obstructive pulmonary disease exacerbation, severe renal failure (creatinine serum levels of >400 μmol/L), active malignant disease, systemic autoimmune disease, pregnancy, or a patient’s unwillingness to participate led to their exclusion from the study (study flow chart—Scheme S1). Details from the data collection on the patients’ history as well as physical and echocardiography examinations were described in our previous reports [32,33]. In addition to routinely performed laboratory tests, for study purposes, a sample of 36 mL of venous blood was obtained from each study patient at the time they presented to the Emergency Department. All enrolled patients received guideline-directed treatment for AHF, as proposed by the European Society of Cardiology [4]. Study patients were followed up for one year after index AHF hospitalization, and the primary endpoint was all-cause mortality.

Prior to enrolment and any study procedure, written informed consent for participation was obtained from each enrolled patient in compliance with the Good Clinical Practice guidelines, and the investigation conformed with the principles outlined in the Declaration of Helsinki [34]. The study was approved by the local Ethics Committee of the Sisters of Charity University Hospital Centre, Zagreb, Croatia (EP 2258/18-10), and the Medical University of Graz, Austria (EK 33-258 ex 20/21).

### 2.2. Laboratory Analyses and Procedures

Routine laboratory parameters, assessed at the time of the patients’ presentation to the Emergency Department, regardless of their fasting status as per hospital protocols for AHF management, included creatinine, estimated glomerular filtration rate (eGFR), blood urea nitrogen, sodium, potassium, chloride, serum glucose, total protein, albumin, total cholesterol, high-density lipoprotein cholesterol (HDL-C), low-density lipoprotein cholesterol (LDL-C), triglycerides, C-reactive protein (CRP), bilirubin, alanine aminotransferase (ALT), aspartate aminotransferase (AST), lactate dehydrogenase (LDH), creatine kinase (CK), high-sensitivity troponin I (hsTnI), N-terminal pro-brain natriuretic peptide (NT-proBNP), blood cell counts, and hemoglobin, fibrinogen, prothrombin time, and international normalized ratio (INR), as well as arterial blood gases. Laboratory procedures and equipment used for assessment of the abovementioned parameters have been described in detail in our previous reports [32]. All additional laboratory procedures and analyses for study purposes were performed using the stored samples of the patients’ sera at the Gottfried Schatz Research Center, Medical University of Graz, Graz, Austria.

### 2.3. Quantification of FFAs

All solvents and reagents were from Sigma-Aldrich (St. Louis, MO, USA). To quantify FFAs, 190 µL of serum was subjected to a Folch extraction (4 mL of chloroform/methanol at 2:1 ratio and 2 mL of water) on a rotating wheel (45 min at ambient temperature). Samples were then centrifuged (500× *g*, 10 min, 4 °C), the lower phase was removed, and the aqueous phase was reextracted. The organic phases were combined, dried under a stream of N_2_, transferred to autosampler vials, and stored under argon at −80 °C. To isolate serum FFAs, the total lipid fraction was subjected to thin-layer chromatography (TLC, unmodified Silica Gel 60 (Merck, Darmstadt, Germany) developed in hexane/diethylether/acetic acid; 70:30:1. The silica region comigrating with oleic acid (spotted on one lane as the FFA standard, stained with iodine vapor) was scraped off, transferred to conical screw-capped derivatization vials, spiked with pentadecanoic acid (C15; 10 µg of internal standard for GC quantitation), and directly methylated (toluene, 1.2 mL; boron trifluoride/methanol complex, 20%; 1 mL; 60 min at 110 °C, sand bath) in the presence of the TLC adsorbent. The reaction was stopped with water (2 mL); fatty acid methyl esters (FAMEs) were extracted with hexane (200 µL), transferred to autosampler vials, dried under N_2_, and redissolved in toluene (25 µL). The FAMEs (1 µL) were analyzed on a Thermo Trace GC Ultra gas chromatograph (Thermo Fisher Scientific, Vienna, Austria) with flame ionization detection (FID). Samples were separated on a 25 m × 0.32 mm FFAP-CB column (Agilent, Vienna, Austria) using the following GC conditions: injector 230 °C, detector 250 °C, with ramping starting at 150 °C, 2.5 °C/min, 215 °C, 10 min, 10 °C/min, and 230 °C, 12.5 min. Fatty acid concentrations were quantified via peak area comparison with the internal standard (C15) and converted to µmol/L. FAMEs prepared from a control serum were run after every 23rd patient sample (the daily throughput) as a laboratory control sample.

### 2.4. Statistics

Metric parameters are summarized as the mean and standard deviation (SD) or median and interquartile range, whereas absolute and relative frequencies were used to describe categorical parameters. Differences between groups defined by various clinical characteristics were tested with the Mann–Whitney U test. The Spearman correlation coefficient was used to assess correlations between FFAs and various clinical and laboratory parameters. Results are presented in a heatmap. A *p*-value < 0.005 (0.05/10) was considered significant after a Bonferroni correction for multiple testing due to the 10 investigated FFA parameters. Our data allow for the detection of significant group differences between alive and deceased patients within 12 months (n = 190 vs. n = 114) for effect sizes of at least 0.39 or for correlation coefficients within the whole cohort (n = 304) of at least 0.19, with a power of 90%. R version 4.1.0 was used for these analyses.

## 3. Results

### 3.1. Patients’ Clinical Characteristics, Chronic Medication, and Standard Laboratory Parameters

A total of 315 patients hospitalized due to AHF were enrolled in the study. The baseline characteristics, comorbidities, chronic medication, and routine laboratory parameters of the whole cohort have been described in our previous reports [32,33].

Since not all serum samples were available for FFA quantification, the following analyses and presented results are based on the data collected from 304 participants.

The patients’ mean (±standard deviation) age was 74.3 ± 10.5 years, and 131 (43.1%) were female. As 281 (92.4%) patients suffered from previously known cardiomyopathy (secondary ischemic, valvular, toxic etiology, primary dilated cardiomyopathies), their present episode of AHF was classified as worsening of chronic disease, while only 23 (7.6%) patients presented with new-onset AHF. The dominant cause of the new-onset AHF was acute myocardial infarction (17/23, 73.9%; 11 with anteroseptal localization and 6 with inferoposterior/posterolateral localization). Only three (1.0%) of the enrolled patients suffered from isolated right ventricle failure (after disqualification of participation for dyspneic patients with chronic pulmonary diseases), while the rest (301, 99.0%) had signs and symptoms of left-sided or global (combination of left- and right-sided) HF. Of the latter, 55 patients (18.3%) presented with pulmonary edema (out of which 60% were hypertensive forms), while the remaining 246 (81.7%) patients with left-sided or global HF had less severe signs of lung congestion. Four patients (1.3%) suffered from cardiogenic shock at the time of presentation to the Emergency Department. According to NYHA classification, the majority of the enrolled patients (288, 94.7%) had dyspnea and tachypnea at rest (NYHA IV), while the others (16, 5.3%) were asymptomatic at rest but could not tolerate minimal physical effort (NYHA III) at the time of presentation. The patients’ comorbidities, vital signs, and other clinical signs of HF, as well as significant echocardiographic measures and routine laboratory test results obtained at the time of presentation to the Emergency Department, are shown in Table 1. Chronic medication is listed in Table S1. One hundred and fourteen (37.5%) patients died within one year after index AHF hospitalization.

### 3.2. Levels of FFAs in Serum of AHF Patients

We determined the concentrations of nine individual FFAs and the sum of these in the serum of 304 AHF patients. The most abundant FFAs were 18:1, 16:0, 18:2, and 18:0. Markedly lower concentrations were found for FFA 16:1, followed by 20:4 and 18:3, whereas the lowest concentrations were found for FFAs 22:6 and 20:5 (Table 2).

### 3.3. Associations of Serum FFAs with Clinical and Laboratory Parameters in AHF Patients

**Figure 1:**
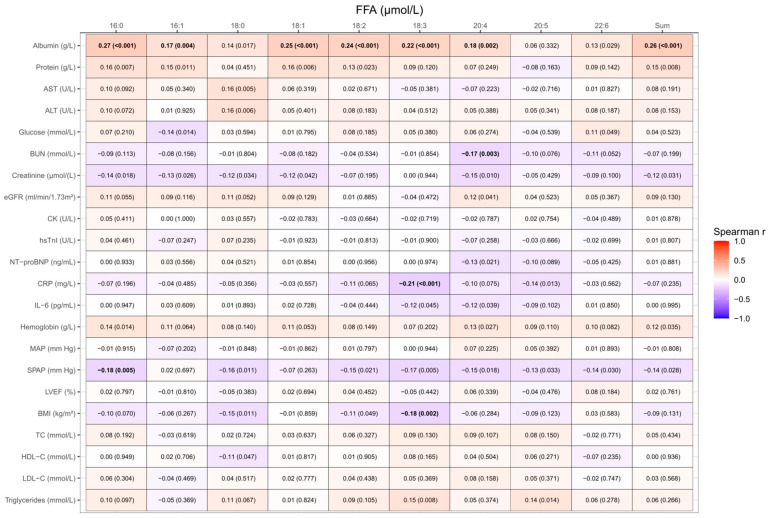
Heatmap of correlation analyses between FFA and the laboratory and clinical parameters of patients with AHF. Values presented are the Spearman correlation coefficients. *p*-values < 0.005 after a Bonferroni correction for multiple testing are considered significant, and significant correlations are depicted in bold. Albumin, protein, and LVEF were measured in 293 patients, SPAP was measured in 253 patients, BUN in 303, and hsTnI in 301 patients; otherwise, the analyses are based on 304 samples. ALT, alanine aminotransferase; AST, aspartate aminotransferase; BMI, body mass index; BUN, blood urea nitrogen; CK, creatin kinase; CRP, C-reactive protein; eGFR, estimated glomerular filtration rate; HDL-C, high-density lipoprotein cholesterol; hsTnI, high-sensitivity troponin I; IL-6, interleukin-6; LDL-C, low-density lipoprotein cholesterol; LVEF, left ventricular ejection fraction; MAP, mean arterial pressure; NT-proBNP, N-terminal pro-brain natriuretic peptide; SPAP, systolic pulmonary pressure; TC, total cholesterol.

As shown in Figure 1, after a Bonferroni correction for multiple testing, the individual FFAs 16:0, 16:1, 18:1, 18:2, 18:3, and 20:4, as well as the total FFAs, were significantly positively correlated with serum albumin levels, albeit not very strongly. Additionally, FFA 16:0 was significantly negatively correlated with systolic pulmonary artery pressure (SPAP), and FFA 20:4 was significantly negatively correlated with blood urea nitrogen (BUN), whereas FFA 18:3 was significantly negatively correlated with C-reactive protein (CRP) and body mass index (BMI). We found no significant correlations of any individual FFA or total FFAs with N-terminal pro-brain natriuretic peptide (NT-proBNP), left ventricular ejection fraction (LVEF), mean arterial pressure (MAP), hemoglobin, liver transaminases, creatine kinase (CK), glucose, creatinine, and estimated glomerular filtration rate (eGFR), as well as interleukin-6 (IL-6) and serum lipid parameters (Figure 1).

### 3.4. Differences in FFAs in Various Groups of AHF Patients

We also examined whether the serum levels of individual and total FFAs differed in groups of AHF patients defined by various clinical characteristics and survival statuses. The results are shown in Tables S2 and S3. After Bonferroni correction, the serum levels of FFA 16:1 but not of the other individual or total FFAs were significantly lower in AHF patients with type 2 diabetes mellitus (T2D) and in AHF patients with coronary artery disease (CAD) compared with AHF patients without these comorbidities. In contrast, the serum levels of individual and total FFAs were similar in AHF patients with metabolic syndrome (MetS), atrial fibrillation (AF), and sign(s) of venous volume overload compared with AHF patients without these comorbidities or sign(s), as well as in AHF patients with new-onset AHF compared with those with AHF that developed on top of CHF. The serum levels of the individual and total FFAs were also similar in AHF patients who were alive and those who died during index AHF hospitalization or within 3 or 12 months after hospitalization due to AHF.

## 4. Discussion

Increased serum levels of natriuretic peptides, catecholamines, and inflammatory cytokines are major promoters of insulin resistance, catabolic dominance, and lipolysis in HF. High-circulating FFAs facilitate cardiac uptake and utilization of FFAs as energy substrates, as well as the conversion of FFAs into lipotoxic compounds [13,20,32]. Deranged cardiac energy metabolism and lipotoxicity compromise the contractile function of the failing heart [13,20,32]. In line with this, elevated total serum FFAs were associated with poor outcomes in AHF patients [26,27], and pharmacological inhibition of cardiac FFA utilization for energy production improved the cardiac function and reduced mortality in AHF patients [22,23].

In contrast to the total serum FFA levels, several studies reported a negative association of serum levels of various individual FFAs with mortality in AHF patients [28,29,30,31]. Low serum levels of FFAs may reflect low adipose tissue lipolysis, increased FFA consumption, or decreased supply due to malnutrition, which is highly prevalent in patients with AHF [33]. Reduced appetite and impaired intestinal absorption, due to gut inflammation, hypoperfusion, and congestion, together with low-grade persistent systemic inflammation, cause malnutrition, wasting, and frailty, the typical features of advanced HF, which are positively associated with unfavorable prognosis in HF patients [33,34].

In the present study, we determined the serum levels of nine individual FFAs and examined the associations of these individual and total FFAs with clinical and laboratory parameters in our AHF patients. In line with the well-established physiological role of albumin as a vehicle for the transport of hydrophobic FFAs, the serum levels of the majority of FFAs were significantly positively correlated with albumin. Since decreased serum albumin reflects a poor nutritional state, impaired intestinal absorption, and reduced biosynthetic capacity of the liver [35], the observed positive associations of FFAs with albumin may suggest a relationship between FFAs, nutritional state, and intestinal and liver function in AHF patients.

Additionally, we found a significant negative association of FFA 16:0 with SPAP and of FFA 20:4 with BUN. These negative associations most likely reflect the regulation of these FFAs, as well as that of SPAP and BUN, in the opposite direction by the underlying AHF pathophysiology. While the HF pathophysiology decreases FFAs levels via the promotion of malnutrition and wasting, the concomitantly worsened renal function (due to hypoperfusion and congestion) increases BUN [8,36], whereas the worsened left ventricular function and consequently increased left ventricular filling pressure increase SPAP [6,37]. Interestingly, these results suggest that FFAs 16:0 and 20:4 do not contribute to the postulated impairment of the heart contractility by toxic derivatives of FFAs in the environment with increased serum levels of FFAs [20]. Several other individual FFAs and the total FFAs were weakly negatively associated with SPAP; however, after a Bonferroni correction, these associations did not remain statistically significant. The significant negative association of FFA 18:3 with CRP that was observed in the present study is in accordance with an inverse association of dietary intake of FFA 18:3 with CRP, as described in a previous study [38]. Although numerous studies reported on the cardiovascular protective effects and anti-inflammatory activities of FFAs 20:5 and 22:6, these FFAs were not significantly associated with CRP or IL-6 in the present study [39,40]. In line with the reported negative association between various plasma FFAs with BMI (reviewed in [41]), in the present study, FFA 18:3 was significantly negatively associated with BMI. Metabolic constellations in adipose tissue and low-grade persistent inflammation have been shown to be potent modulators of desaturases and elongases, the enzymes which catalyze the conversion of FFA 18:3 into FFAs 20:5 and 22:6 [41]. Additionally, a higher BMI may be accompanied by a higher demand for FFAs as energy substrates or for their incorporation into lipids in various tissues, thus resulting in lower serum levels and negative associations of these FFAs with BMI. Several other individual FFAs also showed a weak negative association with BMI, although this did not reach statistical significance.

In the present study, in which 94.7% of patients belonged to New York Heart Association functional class IV (NYHA IV), total serum FFAs were neither associated (with exception of albumin) with clinical and laboratory parameters nor with mortality of these patients. This is in sharp contrast to the results obtained in other AHF cohorts in which the majority of patients belonged to NYHA class III and where total serum FFAs were significantly associated with several clinical and laboratory parameters, as well as mortality [26,27]. This implies that the pathophysiology in severe AHF, as encountered in NYHA class IV patients, disrupts the associations of serum FFAs with clinical and laboratory indicators of the HF severity, as well as abolishing the prognostic capacity of FFAs.

Previous studies have reported associations of circulating FFAs with various comorbidities associated with HF, such as T2D, CAD, hypertension, or AF [42,43,44,45]. In line with the reported beneficial effect of FFA 16:1 on insulin secretion and systemic insulin sensitivity [46], we observed higher FFA 16:1 serum levels in our AHF patients without T2D compared with those with this comorbidity. Higher FFA 16:1 levels in our AHF patients without CAD, compared with those with CAD, suggest a cardioprotective effect of this FFA. However, although FFA 16:1 has been shown to contribute to an anti-atherogenic serum lipoprotein profile, the impact of FFA 16:1 on the cardiovascular system is still not well understood [47].

It is well established that adipose tissue lipolysis mediated by the sequential action of adipose triglyceride lipase (ATGL), hormone-sensitive lipase, and monoglyceride lipase is essential for the supply of tissues with FFAs from the adipose triacylglycerol (TAG) pool to enable the maintenance of systemic energy metabolism during fasting or physical activity, the conditions accompanied by low circulating glucose levels and a high energy demand [48]. However, excessive activation of adipose tissue lipolysis secondary to increased adrenergic activation or increased serum levels of natriuretic peptides and inflammatory cytokines, as encountered in HF, leads to pathologically increased serum levels and increased tissue uptake of FFAs (reviewed in [49]). Excessive uptake and accumulation of TAG and lipotoxic intermediates in the liver and skeletal muscle activate specific protein kinase C isoforms, leading to impaired insulin signaling and T2D [50,51,52]. Insulin resistance in adipose tissue, which in HF is triggered by persistent low-grade inflammation, attenuates the antilipolytic action of insulin and thus also contributes to an increase in serum FFAs and systemic insulin resistance [53]. The impact of adipose-tissue-released FFAs on metabolic health was also demonstrated in obese mice in which adipose-tissue-specific genetic inactivation of ATGL, a master regulator of adipose tissue lipolysis, reduced plasma FFAs and hepatic lipid content, eventually resulting in an improved insulin sensitivity [54,55]. In the present study, however, the serum levels of total FFAs were not correlated with serum glucose levels and were similar in AHF patients with T2D compared with those without this comorbidity. This is most likely due to the severe HF pathophysiology, which disrupts the associations between FFAs and glucose metabolism in our AHF patients.

There are several limitations to the present study: Due to the design, we could not examine causality for the relationship of FFAs with clinical and laboratory parameters. Since the nutritional state of our patients was unknown, we could not examine the impact of fasting/feeding on FFA levels and their associations with clinical and laboratory parameters. Considering the fact that almost 95% of our AHF patients belonged to NYHA class IV, our results do not reflect associations of FFAs with clinical and laboratory parameters in less severe AHF patients.

## 5. Conclusions

We conclude that individual and total serum FFAs are only slightly associated with established clinical and laboratory indicators of HF severity in patients with severe AHF (NYHA class IV). Therefore, measurements of individual or total FFAs would not improve the estimation of the disease severity or risk in NYHA class IV AHF patients.

## Figures and Tables

**Table 1:** Baseline characteristics and laboratory data of AHF patients.

	Data For All Patients with FFA (n = 304)
**Demographics**	
Age (years)	74.3 (10.5)
Sex, female	131 (43.1%)
**Comorbidities**	
Hypertension	283 (93.1%)
T2DM	128 (42.1%)
CAD	152 (50.0%)
CMP	281 (92.4%)
AF	167 (54.9%)
CKD	139 (45.7%)
MetS	208 (68.4%)
**Physical measures at admission**	
MAP (mmHg)	100.0 (90.0, 118.8)
Heart rate (beats/min)	100.0 (80.0, 115.5)
Respiratory rate (breaths/min)	28.0 (24.0, 32.5)
BMI (kg/m^2^)	28.0 (25.0, 31.7)
**Signs and symptoms**	
Symptom duration (days)	5.0 (4.0, 5.0)
Rales or crackles	300 (98.7%)
JVD	165 (54.3%)
Enlarged liver	171 (56.2%)
Ascites	47 (15.5%)
Peripheral edema	198 (65.1%)
**NYHA class**	
3	16 (5.3%)
4	288 (94.7%)
**AHF type**	
New-onset AHF	23 (7.6%)
AHF following CHF	281 (92.4%)
**Echocardiography**	
LVEDd/BSA (mm/m^2^)	28.6 (25.5, 31.8)
LVEF (%)	40.0 (30.0, 50.0)
SPAP (mmHg)	50.0 (45.0, 60.0)
**AHF class**	
HFrEF, EF **<** 40%	138 (47.1%)
HFmrEF, EF 41-49%	80 (27.3%)
HFpEF, EF ≥ 50%	75 (25.6%)
**Laboratory test results at admission**	
TC (mmol/L)	3.5 (2.9, 4.5)
HDL-C (mmol/L)	1.1 (0.9, 1.3)
LDL-C (mmol/L)	1.9 (1.4, 2.7)
Triglycerides (mmol/L)	1.0 (0.8, 1.3)
Albumin (g/L)	37.9 (34.9, 41.3)
Total proteins (g/L)	67.0 (61.0, 71.0)
Bilirubin (µmol/L)	17.3 (11.2, 28.5)
AST (U/L)	27.0 (20.0, 44.2)
ALT (U/L)	25.0 (15.0, 41.2)
Glucose (mmol/L)	7.8 (6.1, 11.1)
Sodium (mmol/L)	140.0 (137.0, 142.0)
Potassium (mmol/L)	4.5 (4.1, 4.8)
Chloride (mmol/L)	103.0 (99.0, 106.0)
BUN (mmol/L)	9.6 (7.0, 14.1)
Creatinine (µmol/L)	117.0 (89.8, 152.0)
eGFR (mL/min/1.73m^2^)	46.3 (32.4, 64.3)
CK (U/L)	93.5 (58.0, 162.2)
LDH (U/L)	264.0 (218.8, 329.2)
hsTnI (ng/L)	46.0 (20.0, 141.0)
NT-proBNP (pg/mL)	6578.5 (3544.5, 15076.2)
CRP (mg/L)	11.8 (5.5, 32.6)
IL-6 (pg/mL)	25.1 (12.9, 59.8)
Fibrinogen (g/L)	4.0 (3.4, 4.7)
Erythrocytes (x 10^12^/L)	4.6 (4.1, 5.0)
Hemoglobin (g/L)	133.5 (119.0, 148.0)
pH	7.4 (7.3, 7.4)
pO_2_ (kPa)	8.8 (7.3, 10.4)
pCO_2_ (kPa)	5.2 (4.5, 6.4)
HCO_3_ (mmol/L)	24.0 (21.3, 27.5)

Data are presented as n (%), mean and standard deviation, or as median and interquartile range (q1, q3). AF, atrial fibrillation; AHF, acute heart failure; ALT, alanine aminotransferase; AST, aspartate aminotransferase; BMI, body mass index; BUN, blood urea nitrogen; CAD, coronary artery disease; CHF, chronic heart failure; CK, creatine kinase; CKD, chronic kidney disease; CMP, cardiomyopathy; CRP, C-reactive protein; EF, ejection fraction; eGFR, estimated glomerular filtration rate; HDL-C, high-density lipoprotein cholesterol; HFrEF, heart failure with reduced ejection fraction; HFmrEF, heart failure with mildly reduced ejection fraction; HFpEF, heart failure with preserved ejection fraction; hsTnI, high-sensitivity troponin I; HDL-C, high-density lipoprotein cholesterol; JVD, jugular vein distension; LDH, lactate dehydrogenase; LDL-C, low-density lipoprotein cholesterol; LVEDd, left ventricle end-diastolic diameter; LVEF, left ventricular ejection fraction; MAP, mean arterial pressure; MetS, metabolic syndrome; NT-proBNP, N-terminal pro-brain natriuretic peptide; NYHA, New York Heart Association Functional Classification; pO_2_, partial oxygen pressure; pCO_2_, partial carbon dioxide pressure; SPAP, systolic pulmonary artery pressure; TC, total cholesterol; T2DM, diabetes mellitus type 2.

**Table 2:** FFA levels in AHF patients (N = 304).

FFA	Serum Levels (µmol/L)
16:0	260.5 (195.9, 310.6)
16:1	25.9 (15.9, 39.8)
18:0	122.8 (97.9, 153.9)
18:1	304.6 (221.8, 404.9)
18:2	150.5 (100.8, 203.6)
18:3	5.1 (3.5, 7.7)
20:4	7.2 (5.3, 10.6)
20:5	0.3 (0.2, 0.7)
22:6	1.2 (0.8, 1.7)
Sum	896.8 (667.3, 1126.2)

Data are presented as median and interquartile range (q1, q3). FFA, free fatty acid.

## Data Availability

Data are available within the article and Supplementary Materials.

## Supplementary Materials

The following supporting information can be downloaded at: https://www.mdpi.com/article/10.3390/biomedicines11123197/s1. Scheme S1: study flow chart; Table S1: chronic medication of AHF patients prior to index AHF hospitalization; Table S2: serum levels of FFAs in various groups of AHF patients; Table S3: differences in FFA levels between AHF patients who were alive and those who died during index AHF hospitalization or within 3 or 12 months after index AHF hospitalization.
