# Supplementary material for: Association between Serum Free Fatty Acids and Clinical and Laboratory Parameters in Acute Heart Failure Patients
Source: Biomedicines. 2023 Dec 1;11(12):3197. doi: 10.3390/biomedicines11123197 (PMC10740773; doi:10.3390/biomedicines11123197)
Supplement: Supplementary file 1 [file biomedicines-11-03197-s001.zip › Table S1.pdf]

Table S1. Chronic medication of AHF patients prior to index AHF hospitalization.

Drug	All patients with FFA data (n=304)
Furosemide	188 (61.8%)
Chlortalidone	22 (7.2%)
Indapamide	21 (6.9%)
MRA	66 (21.7%)
Digoxin	27 (8.9%)
Nitrate	39 (12.8%)
Amiodarone	25 (8.2%)
Beta-blockers	181 (59.5%)
ACEI	160 (52.6%)
Ang II receptor antagonist	32 (10.5%)
Amlodipine	88 (28.9%)
Statins	116 (38.2%)
Insulin	49 (16.1%)
Metformin	51 (16.8%)
Sulphonylurea	33 (10.9%)
ASA	81 (26.6%)
Warfarine	82 (27.0%)

Data are presented as N (%).

ACEI, angiotensin-converting-enzyme inhibitor; AHF, acute heart failure; Ang II, angiotensin II; ASA, acetylsalicylic acid; MRA, mineralocorticoid receptor antagonist.
