# Supplementary material for: Association between Serum Free Fatty Acids and Clinical and Laboratory Parameters in Acute Heart Failure Patients
Source: Biomedicines. 2023 Dec 1;11(12):3197. doi: 10.3390/biomedicines11123197 (PMC10740773; doi:10.3390/biomedicines11123197)
Supplement: Supplementary file 1 [file biomedicines-11-03197-s001.zip › Table S2.pdf]

Table S2. Serum levels of FFAs in various groups of AHF patients.

		**FFA (µmol/L)**									
		**16:0**	**16:1**	**18:0**	**18:1**	**18:2**	**18:3**	**20:4**	**20:5**	**22:6**	**Sum**
T2D	No (N=176)	263.0 (195.1, 317.4)	31.0 (18.1, 47.3)	124.1 (98.2, 152.3)	324.1 (220.8, 419.3)	144.0 (90.6, 203.6)	5.1 (3.5, 7.6)	7.2 (5.4, 10.6)	0.4 (0.2, 0.7)	1.1 (0.8, 1.6)	916.9 (656.3, 1146.9)
	Yes (N=128)	259.4 (195.9, 304.4)	20.9 (13.9, 33.3)	120.1 (97.9, 155.9)	289.2 (224.7, 388.2)	157.5 (108.2, 200.8)	5.2 (3.4, 7.9)	7.2 (5.2, 10.7)	0.2 (0.2, 0.7)	1.2 (0.9, 1.7)	885.1 (678.4, 1094.1)
		p=0.766	**p<0.001**	p=0.925	p=0.192	p=0.310	p=0.692	p=0.997	p=0.621	p=0.261	p=0.510
CAD	No (N=152)	256.5 (190.5, 312.5)	30.5 (17.2, 44.6)	120.7 (97.4, 144.9)	321.3 (216.6, 404.4)	147.1 (90.6, 197.4)	5.0 (3.4, 7.0)	7.2 (5.1, 10.5)	0.3 (0.2, 0.6)	1.2 (0.8, 1.7)	893.1 (635.6, 1129.2)
	Yes (N=152)	262.0 (197.0, 309.3)	23.0 (14.5, 36.0)	125.8 (99.8, 158.3)	289.9 (230.5, 405.6)	157.3 (107.6, 206.7)	5.5 (3.7, 8.3)	7.1 (5.5, 10.7)	0.3 (0.2, 0.8)	1.2 (0.8, 1.6)	900.8 (696.8, 1122.4)
		p=0.586	**p=0.004**	p=0.051	p=0.989	p=0.249	p=0.128	p=0.510	p=0.543	p=0.964	p=0.635
MetS	No (N=98)	260.1 (200.7, 310.9)	27.2 (17.9, 43.6)	123.8 (94.5, 151.3)	299.0 (222.7, 403.6)	143.0 (93.8, 203.2)	5.4 (3.7, 7.6)	6.9 (5.0, 10.8)	0.3 (0.2, 0.7)	1.1 (0.7, 1.5)	904.0 (656.3, 1129.2)
	Yes (N=208)	260.5 (195.5, 310.6)	24.6 (14.7, 39.1)	121.4 (99.0, 155.4)	309.6 (221.5, 405.6)	155.8 (102.9, 206.6)	5.1 (3.5, 7.7)	7.3 (5.4, 10.6)	0.3 (0.2, 0.7)	1.2 (0.9, 1.7)	895.7 (678.4, 1119.1)
		p=0.768	p=0.058	p=0.817	p=0.927	p=0.408	p=0.701	p=0.448	p=0.933	p=0.044	p=0.911
AF	No (N=137)	256.5 (185.0, 309.0)	24.0 (16.0, 39.8)	120.2 (95.4, 158.1)	297.9 (210.2, 427.8)	157.5 (95.0, 206.6)	5.2 (3.5, 8.3)	7.7 (5.7, 10.8)	0.4 (0.2, 0.8)	1.1 (0.8, 1.7)	873.4 (636.0, 1153.9)
	Yes (N=167)	260.5 (203.6, 313.6)	28.3 (15.7, 39.8)	123.0 (100.4, 148.5)	306.8 (231.1, 402.0)	148.0 (102.8, 203.3)	5.1 (3.5, 7.5)	6.6 (4.9, 10.1)	0.3 (0.2, 0.7)	1.2 (0.8, 1.7)	913.4 (693.1, 1097.0)
		p=0.869	p=0.570	p=0.970	p=0.946	p=0.873	p=0.406	p=0.043	p=0.427	p=0.645	p=0.979
Sign(s) *	No (N=65)	268.5 (185.8, 322.2)	22.4 (13.3, 36.1)	122.9 (91.5, 162.0)	285.3 (218.3, 439.6)	158.0 (113.1, 226.9)	5.4 (3.6, 8.3)	7.0 (4.5, 11.1)	0.2 (0.2, 0.6)	1.0 (0.7, 1.5)	918.5 (631.0, 1208.4)
	Yes (N=239)	259.9 (196.9, 306.8)	26.8 (17.1, 40.2)	122.8 (99.3, 152.5)	306.8 (222.7, 404.5)	148.2 (96.8, 196.6)	5.1 (3.5, 7.7)	7.3 (5.4, 10.6)	0.4 (0.2, 0.7)	1.2 (0.8, 1.7)	893.6 (683.4, 1113.1)
		p=0.504	p=0.119	p=0.862	p=0.989	p=0.232	p=0.822	p=0.651	p=0.114	p=0.135	p=0.681
AHF type	New onset AHF (N=23)	278.0 (187.2, 330.4)	23.7 (13.9, 31.6)	116.3 (98.7, 158.1)	316.4 (227.1, 389.6)	122.7 (87.0, 222.7)	4.4 (3.0, 9.2)	7.2 (4.0, 10.7)	0.2 (0.2, 0.6)	1.0 (0.7, 2.0)	912.1 (625.7, 1195.3)
	AHF following CHF (N=281)	259.9 (195.9, 309.7)	26.4 (16.3, 40.3)	122.9 (97.9, 153.1)	302.3 (221.8, 404.7)	151.8 (101.9, 203.1)	5.3 (3.6, 7.6)	7.2 (5.4, 10.6)	0.3 (0.2, 0.7)	1.2 (0.8, 1.7)	895.8 (670.2, 1121.2)
		p=0.806	p=0.226	p=0.940	p=0.814	p=0.535	p=0.362	p=0.497	p=0.205	p=0.586	p=0.897

Data are presented as median and interquartile range (q1, q3). Differences in FFA levels between the groups were tested with the Mann-Whitney U test.
P-values < 0.005 are considered significant after a Bonferroni correction for multiple testing and are depicted in bold.
AF, atrial fibrillation; AHF, acute heart failure; CAD, coronary artery disease; CHF, chronic heart failure; FFA, free fatty acid; MetS, metabolic syndrome; T2D, type 2 diabetes mellitus.
*Any of the following: peripheral edema, enlarged liver, ascites or jugular venous distension.
