# Supplementary material for: Association between Serum Free Fatty Acids and Clinical and Laboratory Parameters in Acute Heart Failure Patients
Source: Biomedicines. 2023 Dec 1;11(12):3197. doi: 10.3390/biomedicines11123197 (PMC10740773; doi:10.3390/biomedicines11123197)
Supplement: Supplementary file 1 [file biomedicines-11-03197-s001.zip › Table S3.pdf]

Table S3. Differences in FFA levels between AHF patients who were alive and those who died during index AHF hospitalization or within 3 or 12 months after index AHF hospitalization.

**Index AHF hospitalization**				
**(μmol/L)**	**Alive (N=271)**	**Deceased (N=33)**	**Total (N=304)**	**p-value**
FFA 16:0	261.6 (193.2, 311.0)	260.5 (222.5, 304.1)	260.5 (195.9, 310.6)	0.889
FFA 16:1	24.8 (15.2, 39.8)	27.4 (19.8, 46.7)	25.9 (15.9, 39.8)	0.440
FFA 18:0	122.8 (97.8, 154.0)	122.8 (98.4, 152.9)	122.8 (97.9, 153.9)	0.792
FFA 18:1	302.3 (217.5, 405.6)	307.3 (243.2, 371.3)	304.6 (221.8, 404.9)	0.951
FFA 18:2	150.3 (101.7, 204.8)	151.8 (100.7, 195.4)	150.5 (100.8, 203.6)	0.874
FFA 18:3	5.3 (3.5, 7.7)	4.6 (3.6, 6.1)	5.1 (3.5, 7.7)	0.452
FFA 20:4	7.3 (5.4, 10.7)	6.5 (5.1, 8.0)	7.2 (5.3, 10.6)	0.083
FFA 20:5	0.4 (0.2, 0.7)	0.2 (0.2, 0.4)	0.3 (0.2, 0.7)	0.067
FFA 22:6	1.2 (0.8, 1.7)	1.3 (0.9, 1.6)	1.2 (0.8, 1.7)	0.866
Sum	903.8 (643.6, 1129.4)	893.0 (757.2, 1112.9)	896.8 (667.3, 1126.2)	0.957
**3 months after index AHF hospitalization**				
**(μmol/L)**	**Alive (N=233)**	**Deceased (N=71)**	**Total (N=304)**	**p-value**
FFA 16:0	262.0 (188.1, 320.9)	252.6 (216.9, 300.2)	260.5 (195.9, 310.6)	0.844
FFA 16:1	24.7 (14.5, 39.8)	27.2 (19.8, 42.9)	25.9 (15.9, 39.8)	0.232
FFA 18:0	123.7 (97.9, 153.8)	120.9 (98.1, 154.1)	122.8 (97.9, 153.9)	0.915
FFA 18:1	311.9 (210.8, 407.8)	296.7 (240.7, 401.2)	304.6 (221.8, 404.9)	0.760
FFA 18:2	157.1 (98.8, 210.4)	145.9 (102.1, 186.1)	150.5 (100.8, 203.6)	0.448
FFA 18:3	5.4 (3.5, 8.1)	4.7 (3.6, 6.0)	5.1 (3.5, 7.7)	0.121
FFA 20:4	7.3 (5.5, 10.9)	6.6 (5.1, 8.5)	7.2 (5.3, 10.6)	0.069
FFA 20:5	0.4 (0.2, 0.8)	0.2 (0.2, 0.5)	0.3 (0.2, 0.7)	0.043
FFA 22:6	1.2 (0.8, 1.7)	1.2 (0.9, 1.6)	1.2 (0.8, 1.7)	0.718
Sum	912.2 (636.0, 1142.0)	892.5 (738.1, 1075.7)	896.8 (667.3, 1126.2)	0.893
**12 months after index AHF hospitalization**				
**(μmol/L)**	**Alive (N=190)**	**Deceased (N=114)**	**Total (N=304)**	**p-value**
FFA 16:0	264.7 (199.2, 326.5)	246.7 (186.7, 301.3)	260.5 (195.9, 310.6)	0.169
FFA 16:1	26.7 (15.7, 40.3)	24.7 (16.1, 37.0)	25.9 (15.9, 39.8)	0.555
FFA 18:0	123.8 (100.4, 154.1)	120.2 (95.4, 152.7)	122.8 (97.9, 153.9)	0.453
FFA 18:1	318.3 (216.7, 415.7)	288.9 (231.0, 402.2)	304.6 (221.8, 404.9)	0.629
FFA 18:2	154.5 (103.5, 215.3)	147.0 (100.1, 190.3)	150.5 (100.8, 203.6)	0.264
FFA 18:3	5.6 (3.6, 8.5)	4.7 (3.5, 6.1)	5.1 (3.5, 7.7)	0.018
FFA 20:4	7.6 (5.5, 11.2)	6.6 (5.1, 8.7)	7.2 (5.3, 10.6)	0.015
FFA 20:5	0.4 (0.2, 0.8)	0.2 (0.2, 0.6)	0.3 (0.2, 0.7)	0.027
FFA 22:6	1.2 (0.8, 1.8)	1.2 (0.8, 1.6)	1.2 (0.8, 1.7)	0.380
Sum	921.9 (647.5, 1149.0)	868.0 (685.4, 1085.5)	896.8 (667.3, 1126.2)	0.279

Data are presented as median and interquartile range (q1, q3). Differences in FFA levels between the groups were tested with the Mann-Whitney U test.

P-values < 0.005 are considered significant after a Bonferroni correction for multiple testing.

FFA, free fatty acid.
