# Supplementary figures and images for: Association between Serum Free Fatty Acids and Clinical and Laboratory Parameters in Acute Heart Failure Patients
Source: Biomedicines. 2023 Dec 1;11(12):3197. doi: 10.3390/biomedicines11123197 (PMC10740773; doi:10.3390/biomedicines11123197)

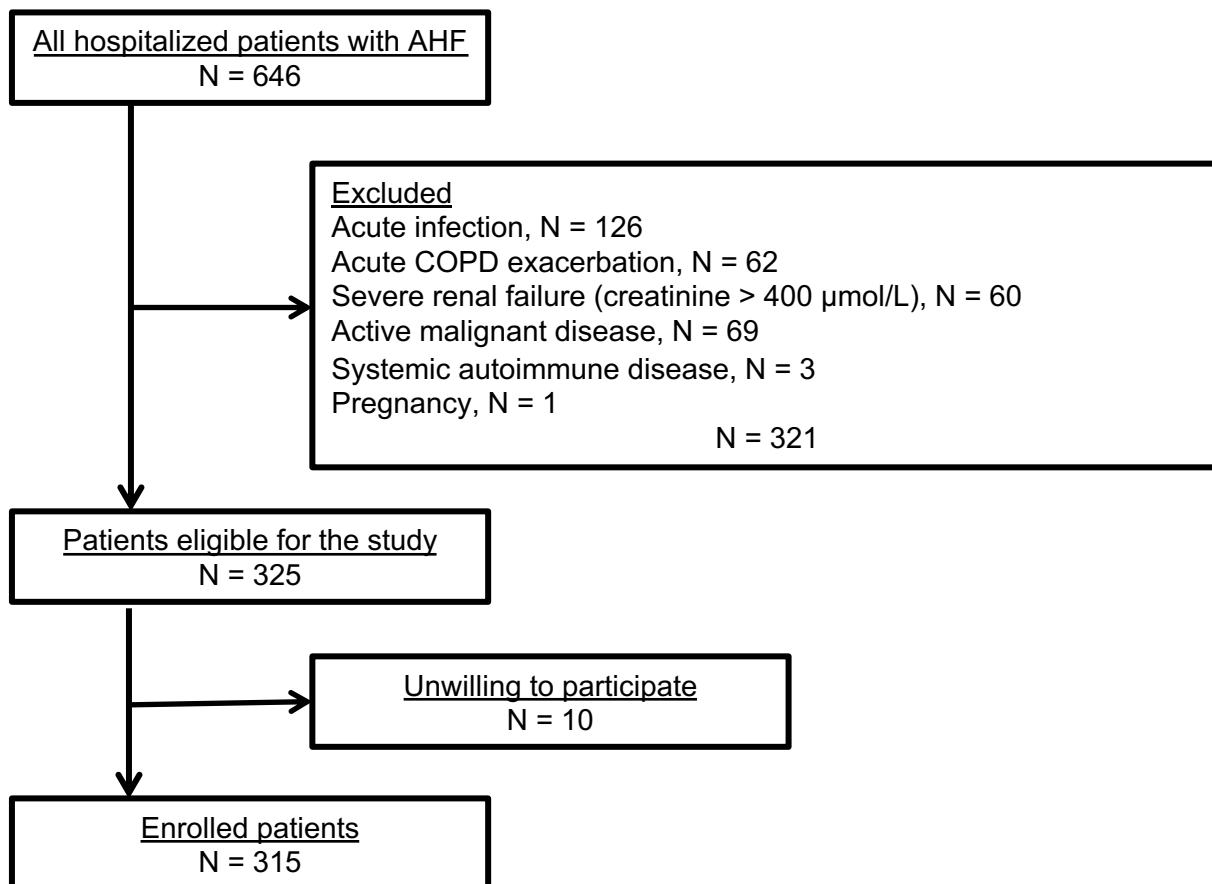

Supplement: Supplementary file 1 [file biomedicines-11-03197-s001.zip › Scheme. S1.pdf]
